# Performance of large language models in medical licensing examinations: a systematic review and meta-analysis

**DOI:** 10.3352/jeehp.2025.22.36

**Published:** 2025-11-18

**Authors:** Haniyeh Nouri, Abdollah Mahdavi, Ali Abedi, Alireza Mohammadnia, Mahnaz Hamedan, Masoud Amanzadeh

**Affiliations:** 1 Student Research Committee, School of Medicine, Ardabil University of Medical Sciences, Ardabil, Iran; 2 Department of Health Information Management, School of Medicine, Ardabil University of Medical Sciences, Ardabil, Iran; 3 Department of Physiology, School of Medicine, Ardabil University of Medical Sciences, Ardabil, Iran; The Catholic University of Korea, Korea

**Keywords:** Artificial intelligence, Large language models, Medical education, Medical examination

## Abstract

**Purpose:**

This study systematically evaluates and compares the performance of large language models (LLMs) in answering medical licensing examination questions. By conducting subgroup analyses based on language, question format, and model type, this meta-analysis aims to provide a comprehensive overview of LLM capabilities in medical education and clinical decision-making.

**Methods:**

This systematic review, registered in PROSPERO and following PRISMA (Preferred Reporting Items for Systematic Reviews and Meta-Analyses) guidelines, searched MEDLINE (PubMed), Scopus, and Web of Science for relevant articles published up to February 1, 2025. The search strategy included Medical Subject Headings (MeSH) terms and keywords related to (“ChatGPT” OR “GPT” OR “LLM variants”) AND (“medical licensing exam*” OR “medical exam*” OR “medical education” OR “radiology exam*”). Eligible studies evaluated LLM accuracy on medical licensing examination questions. Pooled accuracy was estimated using a random-effects model, with subgroup analyses by LLM type, language, and question format. Publication bias was assessed using Egger’s regression test.

**Results:**

This systematic review identified 2,404 studies. After removing duplicates and excluding irrelevant articles through title and abstract screening, 36 studies were included after full-text review. The pooled accuracy was 72% (95% confidence interval, 70.0% to 75.0%) with high heterogeneity (I^2^=99%, P<0.001). Among LLMs, GPT-4 achieved the highest accuracy (81%), followed by Bing (79%), Claude (74%), Gemini/Bard (70%), and GPT-3.5 (60%) (P=0.001). Performance differences across languages (range, 62% in Polish to 77% in German) were not statistically significant (P=0.170).

**Conclusion:**

LLMs, particularly GPT-4, can match or exceed medical students’ examination performance and may serve as supportive educational tools. However, due to variability and the risk of errors, they should be used cautiously as complements rather than replacements for traditional learning methods.

## Graphical abstract

[Fig f3-jeehp-22-36]

## Introduction

### Background

Large language models (LLMs) represent a category of artificial intelligence (AI) systems that have acquired human-like comprehension and reasoning capabilities through transformer architectures [1,2]. By leveraging deep learning and advanced artificial neural networks, these models can interpret relationships between characters and words and generate coherent text. They are trained on billions of parameters to automatically identify complex patterns and relationships within massive datasets [3]. In recent years, LLMs have demonstrated considerable potential across various domains, including programming, commerce, law, translation, and others [4-6]. This technology has also garnered substantial attention in medical sciences and healthcare, and the utilization of these models among students and faculty for health and medical examinations has recently become a prevalent practice [7,8]. These examinations serve as practical benchmarks for assessing model accuracy, and the results can be used for educational purposes and for comparison with healthcare students [9-11]. In recent years, numerous studies have investigated LLM performance on medical licensing examination questions in various languages and countries [12-18]. Nevertheless, despite the promising potential of these models, their application in healthcare contexts faces notable limitations. Given the sensitivity of the field, the generation of incorrect or misleading outputs due to hallucination, misinterpretation, bias, over-reliance, incomplete training, or lack of transparency continues to hinder full trust and adoption [19-22].

### Objectives

Although many primary studies have examined the performance of LLMs across different medical examinations, their findings vary widely depending on model version, specialty domain, and question type. These inconsistencies make it difficult to draw a clear conclusion regarding the overall capabilities of LLMs in medical education. Therefore, a systematic review and meta-analysis is warranted. Previous reviews have often focused on a single model—most notably ChatGPT [4,9,23]—without comparing other emerging systems. In contrast, our meta-analysis evaluates multiple LLMs, including ChatGPT (OpenAI), Gemini (Google), Copilot (Microsoft), and Claude (Anthropic). Furthermore, we conducted subgroup analyses based on language, question format, and content type, which have received limited attention in earlier research. Considering these factors, this meta-analysis provides a comprehensive summary of the performance of 4 widely used LLMs across various health-related examinations, without restrictions by country or language. Ultimately, this study explores the capacity of these models to serve as supportive educational tools and offers insights into their potential application in decision-making within medical education.

## Methods

### Ethics statement

This study was based entirely on previously published literature; therefore, ethical approval and informed consent were not required.

### Study design

**Fig. 1.**
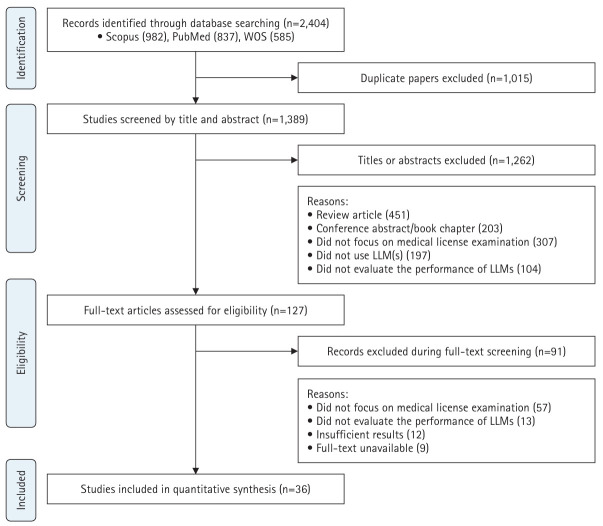
Preferred reporting items PRISMA (Preferred Reporting Items for Systematic Reviews and Meta-Analyses) flow diagram for systematic reviews and meta-analyses. LLM, large language model.

This study followed the Preferred Reporting Items for Systematic Reviews and Meta-Analyses (PRISMA) guidelines (Fig. 1) and was registered in PROSPERO (CRD420251055880).

### Eligibility criteria

Eligibility criteria for study selection were defined based on the PICOS (population, intervention, comparison, outcome, study design) framework (Table 1).

### Information sources

A comprehensive search of electronic databases, including MEDLINE (PubMed), Scopus, and Web of Science (WOS), was performed to identify relevant studies published up to February 1, 2025.

### Search strategy

The search strategy used a combination of Medical Subject Headings (MeSH) terms and relevant keywords, including (“ChatGPT” OR “GPT” OR “Generative Pre-trained Transformer” OR “Gemini” OR “Bard” OR “Claude” OR “Copilot” OR “Bing” OR “large language model*” OR “LLM”) AND (“medical licensing exam*” OR “medical exam*” OR “medical license*” OR “medical education”). The complete search strategy is provided in Supplement 1. All retrieved records were managed in EndNote ver. 20.0 (Clarivate), and duplicates were removed prior to screening.

### Selection process

Two investigators (M.A. and H.N.) independently and in duplicate screened all studies. Titles and abstracts were reviewed first to identify potentially eligible studies, followed by full-text assessments to confirm inclusion. Disagreements were resolved through discussion and consensus; if consensus was not reached, a third reviewer (M.H.) provided the final decision. The screening process was documented, including the number of articles reviewed and reasons for exclusion at each stage (Supplement 2).

### Data collection process

Two reviewers (M.A. and H.N.) independently extracted data from all included studies using a standardized data-extraction form in Microsoft Excel (Microsoft Corp.). Any discrepancies were resolved through discussion, with arbitration by a third reviewer (M.H.) when necessary. The extraction form is available in Supplement 3.

### Data items

The following information was extracted from each study: first author’s name, publication year, country, question language, question source, number of questions, question format, type of LLM used, and LLM accuracy. Accuracy was defined as the percentage of correct responses provided by the LLM out of the total number of examination questions. If accuracy was not explicitly reported, it was calculated by dividing the number of correct answers by the total number of questions. In studies evaluating multiple LLMs, data for each model were collected separately (Dataset 1).

### Study risk of bias assessment

The quality and risk of bias of included studies were evaluated using a modified version of the QUADAS-2 tool (University of Bristol), previously applied in similar research [9]. This adapted framework comprises 21 items across 4 domains: question selection, index model, reference standard, and flow/timing. Two reviewers (M.A. and H.N.) conducted independent assessments, with discrepancies resolved by a third reviewer (M.H.).

### Synthesis methods

A meta-analysis was performed using a random-effects model to estimate the pooled accuracy of LLMs. Forest plots were generated to visualize overall accuracy along with corresponding 95% confidence intervals (CIs). Heterogeneity across studies was assessed using the I^2^ statistic, with values greater than 50% indicating substantial heterogeneity. Subgroup analyses were performed based on LLM type (e.g., GPT-4, GPT-3.5, Gemini), question format (text-based or image-based), and question language (English vs. non-English).

To explore potential sources of heterogeneity, sensitivity analyses were conducted using the leave-one-out method, recalculating pooled accuracy after sequentially excluding each study. Publication bias was evaluated through visual inspection of funnel-plot asymmetry and statistically tested using Egger’s regression test; a significant result indicated potential publication bias. All statistical analyses were performed using the meta and metaprop packages in Stata ver. 17.0 (Stata Corp.).

## Results

### Study selection

In this systematic review, 2,404 studies were identified across multiple databases. After removing duplicates and screening titles and abstracts, 127 articles were selected for full-text review. Following the eligibility assessment, 36 articles met the inclusion criteria and were included in the final analysis. The study selection process is presented in Fig. 1.

### Study characteristics

The characteristics of the included studies are summarized in Table 2. The studies were published between 2023 and 2024, with 21 appearing in 2024 and 15 in 2023. They originated from various countries, including Germany (n=5) [12,16,24-27], Japan (n=5) [18,28-31], Poland (n=5) [14,32-35], USA (n=4) [36-39], China (n=4) [17,40-43], Peru (n=2) [44,45], Saudi Arabia (n=2) [46,47], Taiwan (n=2) [48,49], Brazil (n=1) [23], UK (n=1) [50], Australia (n=1) [15], Belgium (n=1) [51], Chile (n=1) [52], Iran (n=1) [53], and Spain (n=1) [13]. Regarding the language of the questions, most studies used English (n=11), while others used Chinese (n=6), Japanese (n=5), Polish (n=5), Spanish (n=4), German (n=2), Portuguese (n=1), Arabic (n=1), and Italian (n=1).

The number of questions per study ranged from 95 to 2,700. Twenty-seven studies used text-based questions exclusively, while 9 combined text and images. In terms of question format, 31 studies employed multiple-choice questions (MCQs), 3 used single-choice questions (SCQs), and 2 incorporated both MCQs and SCQs. Further study details are provided in Supplement 4.

### Risk of bias in studies

Using the QUADAS-2 method, we evaluated study quality and potential bias. Most studies demonstrated a low risk of bias across all domains; however, some uncertainties were noted in the index test and flow/timing domains. Detailed quality assessment results are provided in Supplements 5 and 6.

### Accuracy of LLMs

**Fig. 2.**
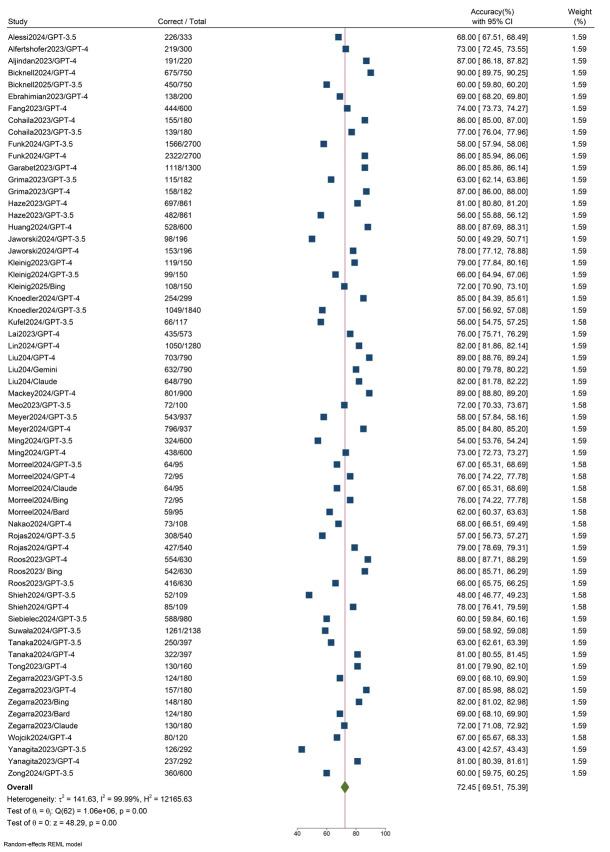
Forest plot for the pooled accuracy of large language models (LLMs). CI, confidence interval.

Across the included studies, 63 different LLMs were evaluated in medical examinations. These included GPT-4 (n=30), GPT-3.5 (n=23), Bard/Gemini (n=3), Claude (n=3), and Bing (n=4). Reported LLM accuracy ranged from 43% for GPT-3.5 to 90% for GPT-4. The pooled accuracy of all models was 72% (95% CI, 70.0%–75.0%). The heterogeneity across studies was significant (I^2^=99.99%, P<0.001). A forest plot summarizing pooled accuracy is shown in Fig. 2.

### Subgroup analysis of the accuracy of LLMs

Subgroup analyses were conducted based on LLM type, question language, and question format (Table 3). By model type, pooled accuracy was highest for GPT-4 (81%), followed by Bing (79%), Claude (74%), Gemini/Bard (70%), and GPT-3.5 (60%), with statistically significant differences among models (P=0.001). When stratified by question format, studies incorporating both text- and image-based questions achieved slightly higher pooled accuracy compared with text-only studies (78% vs. 71%, P=0.03). In terms of question language, pooled LLM accuracy ranged from 62% to 77%, with the highest accuracy observed for German and the lowest for Polish. These differences, however, were not statistically significant (P=0.17). Detailed subgroup results and corresponding forest plots are provided in Supplements 7–11.

### Sensitivity analysis and publication bias

A sensitivity analysis using a leave-one-out approach was conducted to explore potential sources of heterogeneity. The results indicated that excluding individual studies did not meaningfully alter the pooled accuracy (Supplement 12). Publication bias was assessed through funnel-plot visualization and Egger’s regression test (Supplement 13). Visual inspection revealed no substantial asymmetry, consistent with Egger’s test results showing no evidence of small-study effects, thereby supporting the absence of publication bias (P=0.75).

## Discussion

In this systematic review and meta-analysis, we examined 36 peer-reviewed studies that evaluated the accuracy of 63 LLMs in answering medical licensing examination questions. The analysis encompassed diverse languages, question formats, and examination contexts. The overall pooled accuracy of the models was 72%, reflecting generally strong performance. Among the LLMs assessed, GPT-4, GPT-3.5, Google Gemini/Bard, and Microsoft Bing Chat were the most frequently evaluated. GPT-4 demonstrated the highest pooled accuracy (81%), significantly outperforming GPT-3.5 (60%), Claude (74%), Gemini/Bard (70%), and Bing (79%). A recent study by Liu et al. [9] reported pooled accuracies of 81% for GPT-4 and 58% for GPT-3, findings consistent with our results. In another study, Waldock et al. [10] found a pooled accuracy of 61% for all LLMs and 64% for ChatGPT. The discrepancy may reflect both temporal improvements in model performance and methodological differences—Waldock et al. [10] combined GPT-3.5 and GPT-4 in their estimates, whereas our analysis reports each model separately. The observed heterogeneity (I^2^=99%) underscores the variation in study design, question type, language, and model selection, reflecting real-world complexity but complicating direct comparisons. Subgroup analyses confirmed that model type substantially influences performance, with GPT-4 consistently surpassing earlier models. This trend aligns with ongoing advances in natural language processing and highlights the importance of continuous model refinement for specialized medical applications. Our results support previous findings demonstrating GPT-4’s superior performance in clinical reasoning, discipline-specific tasks, and general test-taking ability [9]. For example, studies in ophthalmology and general medicine have shown that GPT-4 performs at or above the level of medical students when responding to board-style questions [11]. These findings collectively suggest that model architecture, training data scope, and domain-specific tuning play critical roles in enhancing diagnostic and interpretive accuracy in medical contexts.

The subgroup analysis further revealed that incorporating image-based content significantly improved pooled accuracy—78% compared with 71% for text-only formats (P=0.030). This improvement aligns with studies showing that multimodal prompting, particularly in GPT-4, enhances diagnostic reasoning and clinical comprehension [54]. Although LLM performance varied across languages—from 62% in Polish to 77% in German—these differences were not statistically significant, contrasting with earlier reports that emphasized language-related disparities [21,55]. The convergence observed here may reflect improved multilingual training and cross-lingual generalization in recent model architectures, especially GPT-4.

The remarkable accuracy of models such as GPT-4 and Bing underscores their potential as valuable tools in medical education, particularly in resource-limited settings where access to expert instructors is constrained. Their consistent accuracy across languages and question types also suggests the feasibility of global deployment. Furthermore, their ability to process image-based questions highlights new opportunities for training in clinically relevant skills such as radiologic interpretation. Despite these encouraging results, substantial heterogeneity (I^2^>95%) indicates that variations in study design, evaluation criteria, and question sources remain influential. Differences in model versions (e.g., GPT-3.5 vs. GPT-4), question difficulty, and dataset size likely contributed to this variability. The inclusion of both official examination questions and public question banks may also account for part of the heterogeneity observed. These methodological differences should therefore be considered when interpreting pooled estimates.

This study has several limitations. First, the high heterogeneity among studies—likely due to differences in question sources, languages, test formats, and study designs—limits the generalizability of the results. Second, a substantial proportion of the reviewed studies focused on OpenAI models (GPT-3.5 and GPT-4), which may have introduced bias into the analysis. Third, detailed information on prompt design, question difficulty, or the specific model versions used was not available in all studies. Furthermore, because of continuous model updates and new releases, the performance reported at a given point in time may not accurately reflect the future performance of these models. Another limitation is the potential overlap between examination questions and the training data of LLMs. As many of the question banks used in the included studies were publicly available, it is possible that some items were encountered by the models during pretraining, which could have slightly inflated performance estimates. However, our subgroup analysis based on question source showed only a minimal difference in accuracy (71% for internal exams vs. 73% for public databases), suggesting that this effect was limited. Nonetheless, potential data contamination should be taken into account when interpreting the results, particularly for widely trained models such as GPT-4.

Future research should focus on improving LLM architectures for medical reasoning, exploring their ability to directly interpret imaging data, and evaluating their impact on real-world diagnostic workflows. In addition, longitudinal studies examining the integration of LLMs into medical curricula—particularly in simulation-based training and formative assessments—could provide valuable frameworks for advancing the practical application of this technology in medical education. Ultimately, while current LLMs show considerable promise, their integration into medical education and practice must proceed with careful awareness of their limitations, ensuring that AI supports rather than replaces expert human judgment. Ethical considerations and potential challenges associated with these models must also be carefully addressed.

## Conclusion

This systematic review and meta-analysis highlight the promising performance of LLMs, particularly GPT-4, in answering medical licensing examination questions. Our findings indicate that LLMs hold significant potential to enhance medical education, assessment, and clinical decision-making. Despite substantial progress, the high heterogeneity among studies and the intrinsic limitations of LLMs suggest that these technologies are not yet ready to replace traditional educational resources or to be used independently in formal assessments. Therefore, LLMs should be regarded as supportive educational tools that complement conventional learning methods, guided by ethical principles and scientific standards. Future efforts should aim to develop standardized assessment protocols and conduct comparative research in authentic educational and clinical settings to ensure the safe and effective implementation of these technologies.

## Figures and Tables

**Figure f3-jeehp-22-36:**
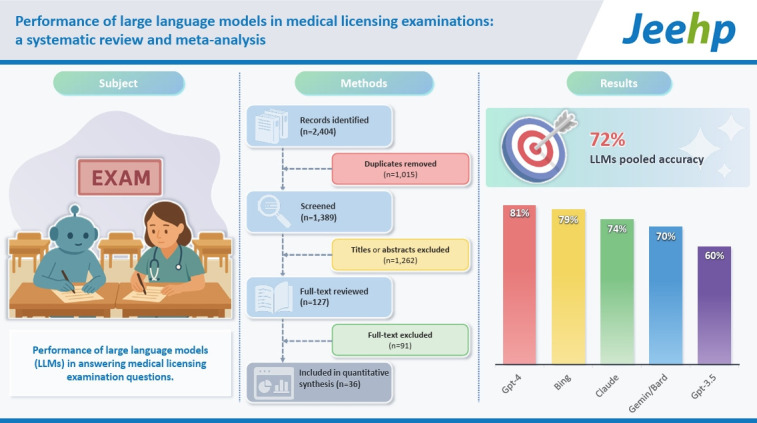


**Table 1.** Inclusion and exclusion criteria based on the PICOS framework

PICOS	Inclusion criteria	Exclusion criteria
Population (P)	Studies assessing the accuracy of LLMs in answering medical license examination questions.	Studies not involving answers to medical license examination questions.
Intervention (I)	Studies that use LLMs (e.g., GPT-4, Gemini, Claude) to answer questions	Research that does not use LLMs.
Comparator (C)	Studies evaluating accuracy of LLM against Human performance (such as medical students) or other AI models	-
Outcome (O)	• Studies reporting accuracy of LLMs	Studies that do not report accuracy or lack sufficient data for metric calculation.
	• Studies that provide data for calculating accuracy (number of correct answers and total number of questions)	
Study design (S)	• Original peer-reviewed studies that examine the performance of LLMs in medical license examinations.	Review, editorials, commentaries, letters to the editor, case series, case reports, conference abstracts and preprint articles
	• Studies published in English language, regardless of the language of the examination questions	

LLMs, large language models; AI, artificial intelligence.

**Table 2.** Characteristics of the included studies

Study (year)	LLMs	Country	Language	Question type	Question format	No. of questions	ACC (%)
Rodrigues Alessi et al. [23] (2024)	GPT-3.5	Brazil	Portuguese	MCQs	Text	333	68
Alfertshofer et al. [24] (2024)	GPT-4	Germany	Italian	MCQs	Text	300	73
Aljindan et al. [46] (2023)	GPT-4	Saudi Arabia	Arabic	MCQs	Text	220	87
Bicknell et al. [36] (2024)	GPT-4	USA	English	MCQs	Text	750	90
	GPT-3.5						60
Ebrahimian et al. [53] (2023)	GPT-4	Iran	English	MCQs	Text	200	69
Fang et al. [40] (2023)	GPT-4	China	Chinese	MCQs	Text	600	74
Flores-Cohaila et al. [44] (2023)	GPT-4	Peru	Spanish	MCQs	Text	180	86
	GPT-3.5						77
Funk et al. [12] (2024)	GPT-3.5	Germany	English	MCQs	Text	2,700	58
	GPT-4						86
Garabet et al. [37] (2024)	GPT-4	USA	English	MCQs	Text	1,300	86
Guillen-Grima et al. [13] (2023)	GPT-3.5	Spain	Spanish	MCQs	Text-image	182	63
	GPT-4						87
Haze et al. [28] (2023)	GPT-4	Japan	Japanese	MCQs–SCQs	Text	861	81
	GPT-3.5						56
Huang et al. [48] (2024)	GPT-4	Taiwan	Chinese	MCQs	Text-image	600	88
Jaworski et al. [14] (2024)	GPT-3.5	Poland	Polish	MCQs	Text	196	50
	GPT-4						78
Kleinig et al. [15] (2023)	GPT-4	Australia	English	MCQs	Text	150	79
	GPT-3.5						66
	Bing						72
Knoedler et al. [25] (2024)	GPT-4	Germany	English	MCQs	Text-image	299	85
	GPT-3.5				Text	1,840	57
Kufel et al. [32] (2024)	GPT-3.5	Poland	Polish	MCQs–SCQs	Text	117	56
Lai et al. [50] (2023)	GPT-4	UK	English	SCQs	Text	573	76
Lin et al. [49] (2024)	GPT-4	Taiwan	Chinese	SCQs	Text-image	1,280	82
Liu et al. [9] (2024)	GPT-4	Japan	Japanese	MCQs	Text-image	790	89
	Gemini						80
	Claude						82
Mackey et al. [38] (2024)	GPT-4	USA	English	MCQs	Text	900	89
Meo et al. [47] (2023)	GPT-3.5	Saudi Arabia	English	MCQs	Text	100	72
Meyer et al. [16] (2024)	GPT-3.5	Germany	German	MCQs	Text-image	937	58
	GPT-4						85
Ming et al. [17] (2024)	GPT-3.5	China	Chinese	MCQs–SCQs	Text	600	54
	GPT-4						73
Morreel et al. [51] (2024)	GPT-3.5	Belgium	English	MCQs	Text	95	67
	GPT-4						76
	Claude						67
	Bing						76
	Bard						62
Nakao et al. [30] (2024)	GPT-4	Japan	Japanese	MCQs	Text-image	108	68
Rojas et al. [52] (2024)	GPT-3.5	Chile	Spanish	MCQs	Text-image	540	57
	GPT-4						79
Roos et al. [27] (2023)	GPT-4	Germany	German	MCQs	Text-image	630	88
	Bing						86
	GPT-3.5						66
Shieh et al. [39] (2024)	GPT-3.5	USA	English	MCQs	Text	109	48
	GPT-4						78
Siebielec et al. [33] (2024)	GPT-3.5	Poland	Polish	MCQs	Text	980	60
Suwała et al. [34] (2024)	GPT-3.5	Poland	Polish	MCQs	Text	2,138	59
Tanaka et al. [18] (2024)	GPT-3.5	Japan	Japanese	MCQs	Text	397	63
	GPT-4						81
Tong et al. [41] (2023)	GPT-4	China	Chinese	SCQs	Text	160	81
Torres-Zegarra et al. [45] (2023)	GPT-3.5	Peru	Spanish	MCQs	Text	180	69
	GPT-4						87
	Bing						82
	Bard						69
	Claude						72
Wojcik et al. [35] (2024)	GPT-4	Poland	Polish	MCQs	Text	120	67
Yanagita et al. [31] (2023)	GPT-3.5	Japan	Japanese	MCQs	Text	292	43
	GPT-4						81
Zong et al. [43] (2024)	GPT-3.5	China	Chinese	MCQs	Text	600	60

LLM, large language model; ACC, accuracy; MCQ, multiple choice question; SCQ, single choice question.

**Table 3.** The results of subgroup analysis

Subgroup	No. (%)	Accuracy (%)	95% CI (%)	I^2^ (%)	P-value	Test of group differences
Overall	63 (100)	72	70.0–75.0	99	0.001	-
LLM types						0.001
GPT-4	30 (48)	81	79.0–83.0	99	0.001	
Bing	4 (6)	79	73.0–85.0	99	0.001	
GPT-3.5	23 (36)	60	57.0–63.0	99	0.001	
Claude	3 (5)	74	65.0–82.0	99	0.001	
Gemini/Bard	3 (5)	70	60.0–81.0	99	0.001	
Question language						0.17
English	21 (33)	72	64.0–77.0	99	0.001	
Chinese	7 (11)	73	64.0–82.0	99	0.001	
German	5 (8)	77	65.0–89.0	99	0.001	
Japanese	10 (16)	72	63.0–81.0	99	0.001	
Polish	6 (10)	62	54.0–69.0	99	0.001	
Spanish	11 (17)	75	69.0–81.0	99	0.001	
Other	3 (5)	76	65.0–87.0	99	0.001	
Question format						0.03
Text	47 (75)	71	67.0–74.0	99	0.001	
Text and image	16 (25)	78	72.0–83.0	99	0.001	
Question type						0.001
MCQ	55 (87)	73	70.0–76.0	99	0.001	
MCQ and SCQ	5 (8)	64	53.0–75.0	99	0.001	
SCQ	3 (5)	72	70.0–75.0	99	0.001	
Source of questions						0.63
Public database	54 (86)	73	69.0–76.0	99	0.001	
Internal exam	9 (14)	71	65.0–77.0	99	0.001	

CI, confidence interval; LLM, large language model; MCQ, multiple choice question; SCQ, single choice question.

## Footnotes

**Data availability**
Data files are available from https://doi.org/10.7910/DVN/KMHVAF Dataset 1. Extracted data for meta-analysis.

## Supplementary materials

Supplement 1. Search strategy for retrieval of publications from databases.

Supplement 2. Excluded studies with reasons.

Supplement 3. Data extraction form.

Supplement 4. Additional details of the included studies.

Supplement 5. Quality assessment of the included studies.

Supplement 6. The detailed information on the results of the quality assessment.

Supplement 7. Subgroup analysis of LLM accuracy based on source of questions.

Supplement 8. Subgroup analysis of LLM accuracy based on different models.

Supplement 9. Subgroup analysis of LLM accuracy based on question language.

Supplement 10. Subgroup analysis of LLM accuracy based on question format.

Supplement 11. Subgroup analysis of LLM accuracy based on question type.

Supplement 12. Leave-one-out meta-analysis.

Supplement 13. Funnel plot for publication bias assessment.

Supplement 14. Audio recording of the abstract.
