# Supplementary material for: Performance of large language models in medical licensing examinations: a systematic review and meta-analysis
Source: J Educ Eval Health Prof. 2025 Nov 18;22:36. doi: 10.3352/jeehp.2025.22.36 (PMC12976628; doi:10.3352/jeehp.2025.22.36)
Supplement: Supplementary file 2 — Supplement 1. Search strategy for retrieval of publications from databases. [file jeehp-22-36-suppl1.docx]

**Supplement 1.** Search strategy for retrieval of publications from databases

| Database | Search terms |
| --- | --- |
| PubMed | (“ChatGPT” OR “GPT” OR “Generative pre-trained transformer” OR “Gemini” OR “Bard” OR “Claude” OR “Copilot” OR “Bing” OR “large language model*” OR “LLM”) AND (“medical licensing exam*” OR “medical exam*” OR “medical license*” OR “medical education”) |
| Scopus | TITLE-ABS-KEY ((“ChatGPT” OR “GPT” OR “Generative pre-trained transformer” OR “Gemini” OR “Bard” OR “Claude” OR “Copilot” OR “Bing” OR “large language model*” OR “LLM”) AND (“medical licensing exam*” OR “medical exam*” OR “medical license*” OR “medical education”)) |
| WOS | TS = ((“ChatGPT” OR “GPT” OR “Generative pre-trained transformer” OR “Gemini” OR “Bard” OR “Claude” OR “Copilot” OR “Bing” OR “large language model*” OR “LLM”) AND (“medical licensing exam*” OR “medical exam*” OR “medical license*” OR “medical education”)) |
