# Supplementary material for: Performance of large language models in medical licensing examinations: a systematic review and meta-analysis
Source: J Educ Eval Health Prof. 2025 Nov 18;22:36. doi: 10.3352/jeehp.2025.22.36 (PMC12976628; doi:10.3352/jeehp.2025.22.36)
Supplement: Supplementary file 3 — Supplement 2. Excluded studies with reasons. [file jeehp-22-36-suppl2.docx]

**Supplement 2.** Data extraction form

| Concept | Description |
| --- | --- |
| Authors | Authors of study |
| Title | Title of publication |
| Publication year | Year of the study was published |
| Study type | Study type |
| Country | The country where the study was published |
| Question language | Language of questions |
| Question source | The source of used questions |
| Question format | The format of questions such as text or image |
| Question type | Multiple choice or single choice |
| Question number | The number of questions which used in study |
| LLM | Type of LLM that used for answering questions |
| Accuracy | Accuracy |
| subspecialties | Subspecialties of questions |

LLM, large language model.
