# Supplementary material for: Performance of large language models in medical licensing examinations: a systematic review and meta-analysis
Source: J Educ Eval Health Prof. 2025 Nov 18;22:36. doi: 10.3352/jeehp.2025.22.36 (PMC12976628; doi:10.3352/jeehp.2025.22.36)
Supplement: Supplementary file 4 — Supplement 3. Data extraction form. [file jeehp-22-36-suppl3.docx]

**Supplement 3.** Excluded studies with reasons

**No focused on medical license examination (57)**

1. Adams LC, Truhn D, Busch F, Dorfner F, Nawabi J, Makowski MR, Bressem KK. Llama 3 challenges proprietary state-of-the-art large language models in radiology board-style examination questions. Radiology 2024;312:e241191. https://doi.org/10.1148/radiol.241191

2. Ali R, Tang OY, Connolly ID, Zadnik Sullivan PL, Shin JH, Fridley JS, Asaad WF, Cielo D, Oyelese AA, Doberstein CE, Gokaslan ZL, Telfeian AE. Performance of ChatGPT and GPT-4 on neurosurgery written board examinations. Neurosurgery 2023;93:1353-1365. https://doi.org/10.1227/neu.0000000000002632

3. Chan J, Dong T, Angelini GD. The performance of large language models in intercollegiate Membership of the Royal College of Surgeons examination. Ann R Coll Surg Engl 2024;106:700-704. https://doi.org/10.1308/rcsann.2024.0023

4. Chen Y, Huang X, Yang F, Lin H, Lin H, Zheng Z, Liang Q, Zhang J, Li X. Performance of ChatGPT and Bard on the medical licensing examinations varies across different cultures: a comparison study. BMC Med Educ 2024;24:1372. https://doi.org/10.1186/s12909-024-06309-x

5. Cheong RC, Pang KP, Unadkat S, Mcneillis V, Williamson A, Joseph J, Randhawa P, Andrews P, Paleri V. Performance of artificial intelligence chatbots in sleep medicine certification board exams: ChatGPT versus Google Bard. Eur Arch Otorhinolaryngol 2024;281:2137-2143. https://doi.org/10.1007/s00405-023-08381-3

6. Cheung BH, Lau GK, Wong GT, Lee EY, Kulkarni D, Seow CS, Wong R, Co MT. ChatGPT versus human in generating medical graduate exam multiple choice questions: a multinational prospective study (Hong Kong S.A.R., Singapore, Ireland, and the United Kingdom). PLoS One 2023;18:e0290691. https://doi.org/10.1371/journal.pone.0290691

7. Cohen A, Alter R, Lessans N, Meyer R, Brezinov Y, Levin G. Performance of ChatGPT in Israeli Hebrew OBGYN national residency examinations. Arch Gynecol Obstet 2023;308:1797-1802. https://doi.org/10.1007/s00404-023-07185-4

8. Collins BR, Black EW, Rarey KE. Introducing AnatomyGPT: a customized artificial intelligence application for anatomical sciences education. Clin Anat 2024;37:661-669. https://doi.org/10.1002/ca.24178

9. Cuthbert R, Simpson AI. Artificial intelligence in orthopaedics: can Chat Generative Pre-trained Transformer (ChatGPT) pass Section 1 of the Fellowship of the Royal College of Surgeons (Trauma & Orthopaedics) examination? Postgrad Med J 2023;99:1110-1114. https://doi.org/10.1093/postmj/qgad053

10. Farhat F, Chaudhry BM, Nadeem M, Sohail SS, Madsen DO. Evaluating large language models for the National Premedical Exam in India: comparative analysis of GPT-3.5, GPT-4, and Bard. JMIR Med Educ 2024;10:e51523. https://doi.org/10.2196/51523

11. Fiedler B, Azua EN, Phillips T, Ahmed AS. ChatGPT performance on the American Shoulder and Elbow Surgeons maintenance of certification exam. J Shoulder Elbow Surg 2024;33:1888-1893. https://doi.org/10.1016/j.jse.2024.02.029

12. Flores-Cohaila JA, Garcia-Vicente A, Vizcarra-Jimenez SF, De la Cruz-Galan JP, Gutierrez-Arratia JD, Quiroga Torres BG, Taype-Rondan A. Performance of ChatGPT on the Peruvian National Licensing Medical Examination: cross-sectional study. JMIR Med Educ 2023;9:e48039. https://doi.org/10.2196/48039

13. Gandhi AP, Joesph FK, Rajagopal V, Aparnavi P, Katkuri S, Dayama S, Satapathy P, Khatib MN, Gaidhane S, Zahiruddin QS, Behera A. Performance of ChatGPT on the India Undergraduate Community Medicine Examination: cross-sectional study. JMIR Form Res 2024;8:e49964. https://doi.org/10.2196/49964

14. Gencer A, Aydin S. Can ChatGPT pass the thoracic surgery exam? Am J Med Sci 2023;366:291-295. https://doi.org/10.1016/j.amjms.2023.08.001

15. Giannos P. Evaluating the limits of AI in medical specialisation: ChatGPT’s performance on the UK Neurology Specialty Certificate Examination. BMJ Neurol Open 2023;5:e000451. https://doi.org/10.1136/bmjno-2023-000451

16. Goodings AJ, Kajitani S, Chhor A, Albakri A, Pastrak M, Kodancha M, Ives R, Lee YB, Kajitani K. Assessment of ChatGPT-4 in Family Medicine Board Examinations using advanced AI learning and analytical methods: observational study. JMIR Med Educ 2024;10:e56128. https://doi.org/10.2196/56128

17. Gritti MN, AlTurki H, Farid P, Morgan CT. Progression of an artificial intelligence chatbot (ChatGPT) for pediatric cardiology educational knowledge assessment. Pediatr Cardiol 2024;45:309-313. https://doi.org/10.1007/s00246-023-03385-6

18. Gummadi R, Dasari N, Kumar DS, Pindiprolu SK. Evaluating the accuracy of large language model (ChatGPT) in providing information on metastatic breast cancer. Adv Pharm Bull 2024;14:499-503. https://doi.org/10.34172/apb.2024.060

19. Haddad F, Saade JS. Performance of ChatGPT on Ophthalmology-related questions across various examination levels: observational study. JMIR Med Educ 2024;10:e50842. https://doi.org/10.2196/50842

20. Hayes DS, Foster BK, Makar G, Manzar S, Ozdag Y, Shultz M, Klena JC, Grandizio LC. Artificial intelligence in orthopaedics: performance of ChatGPT on text and image questions on a complete AAOS Orthopaedic In-Training Examination (OITE). J Surg Educ 2024;81:1645-1649. https://doi.org/10.1016/j.jsurg.2024.08.002

21. Herrmann-Werner A, Festl-Wietek T, Holderried F, Herschbach L, Griewatz J, Masters K, Zipfel S, Mahling M. Assessing ChatGPT’s mastery of Bloom’s taxonomy using psychosomatic medicine exam questions: mixed-methods study. J Med Internet Res 2024;26:e52113. https://doi.org/10.2196/52113

22. Hirano Y, Hanaoka S, Nakao T, Miki S, Kikuchi T, Nakamura Y, Nomura Y, Yoshikawa T, Abe O. GPT-4 Turbo with Vision fails to outperform text-only GPT-4 Turbo in the Japan Diagnostic Radiology Board Examination. Jpn J Radiol 2024;42:918-926. https://doi.org/10.1007/s11604-024-01561-z

23. Hofmann HL, Guerra GA, Le JL, Wong AM, Hofmann GH, Mayfield CK, Petrigliano FA, Liu JN. The rapid development of artificial intelligence: GPT-4’s performance on orthopedic surgery board questions. Orthopedics 2024;47:e85-e89. https://doi.org/10.3928/01477447-20230922-05

24. Hong DR, Huang CY. The performance of AI in medical examinations: an exploration of ChatGPT in ultrasound medical education. Front Med (Lausanne) 2024;11:1472006. https://doi.org/10.3389/fmed.2024.1472006

25. Huang Y, Gomaa A, Semrau S, Haderlein M, Lettmaier S, Weissmann T, Grigo J, Tkhayat HB, Frey B, Gaipl U, Distel L, Maier A, Fietkau R, Bert C, Putz F. Benchmarking ChatGPT-4 on a radiation oncology in-training exam and Red Journal Gray Zone cases: potentials and challenges for ai-assisted medical education and decision making in radiation oncology. Front Oncol 2023;13:1265024. https://doi.org/10.3389/fonc.2023.1265024

26. Huynh LM, Bonebrake BT, Schultis K, Quach A, Deibert CM. New artificial intelligence ChatGPT performs poorly on the 2022 self-assessment study program for urology. Urol Pract 2023;10:409-415. https://doi.org/10.1097/UPJ.0000000000000406

27. Khalpey Z, Kumar U, King N, Abraham A, Khalpey AH. Large language models take on cardiothoracic surgery: a comparative analysis of the performance of four models on American Board of Thoracic Surgery Exam Questions in 2023. Cureus 2024;16:e65083. https://doi.org/10.7759/cureus.65083

28. Khan AM, Sarraf KM, Simpson AI. Enhancements in artificial intelligence for medical examinations: a leap from ChatGPT 3.5 to ChatGPT 4.0 in the FRCS trauma & orthopaedics examination. Surgeon 2025;23:13-17. https://doi.org/10.1016/j.surge.2024.11.008

29. Kim SE, Lee JH, Choi BS, Han HS, Lee MC, Ro DH. Performance of ChatGPT on solving orthopedic board-style questions: a comparative analysis of ChatGPT 3.5 and ChatGPT 4. Clin Orthop Surg 2024;16:669-673. https://doi.org/10.4055/cios23179

30. Kirshteyn G, Golan R, Chaet M. Performance of ChatGPT vs. HuggingChat on OB-GYN topics. Cureus 2024;16:e56187. https://doi.org/10.7759/cureus.56187

31. Kollitsch L, Eredics K, Marszalek M, Rauchenwald M, Brookman-May SD, Burger M, Korner-Riffard K, May M. How does artificial intelligence master urological board examinations?: a comparative analysis of different large language models’ accuracy and reliability in the 2022 In-Service Assessment of the European Board of Urology. World J Urol 2024;42:20. https://doi.org/10.1007/s00345-023-04749-6

32. Kufel J, Bielowka M, Rojek M, Mitręga A, Czogalik L, Kaczynska D, Kondol D, Palkij K, Mielcarska S. Assessing ChatGPT’s performance in national nuclear medicine specialty examination: an evaluative analysis. Iran J Nucl Med 2024;32:60-65. https://doi.org/10.22034/IRJNM.2023.129434.1580

33. Brin D, Sorin V, Vaid A, Soroush A, Glicksberg BS, Charney AW, Nadkarni G, Klang E. Comparing ChatGPT and GPT-4 performance in USMLE soft skill assessments. Sci Rep 2023;13:16492. https://doi.org/10.1038/s41598-023-43436-9

34. Long C, Lowe K, Zhang J, Santos AD, Alanazi A, O’Brien D, Wright ED, Cote D. A novel evaluation model for assessing ChatGPT on otolaryngology-head and neck surgery certification examinations: performance study. JMIR Med Educ 2024;10:e49970. https://doi.org/10.2196/49970

35. Lubitz M, Latario L. Performance of two artificial intelligence generative language models on the Orthopaedic In-Training Examination. Orthopedics 2024;47:e146-e150. https://doi.org/10.3928/01477447-20240304-02

36. Maitland A, Fowkes R, Maitland S. Can ChatGPT pass the MRCP (UK) written examinations?: analysis of performance and errors using a clinical decision-reasoning framework. BMJ Open 2024;14:e080558. https://doi.org/10.1136/bmjopen-2023-080558

37. Nagao T, Yokomizo R, Sekizawa A, Okamoto A. Outstanding performance of ChatGPT on the obstetrics and gynecology board certification examination in Japan: document and image-based questions analysis. J Obstet Gynaecol Res 2024;50:2377-2378. https://doi.org/10.1111/jog.16125

38. Miao J, Thongprayoon C, Cheungpasitporn W, Cornell LD. Performance of GPT-4 Vision on kidney pathology exam questions. Am J Clin Pathol 2024;162:220-226. https://doi.org/10.1093/ajcp/aqae030

39. Mousavi M, Shafiee S, Harley JM, Cheung JCK, Abbasgholizadeh Rahimi S. Performance of generative pre-trained transformers (GPTs) in Certification Examination of the College of Family Physicians of Canada. Fam Med Community Health 2024;12(Suppl 1):e002626. https://doi.org/10.1136/fmch-2023-002626

40. Nakajima N, Fujimori T, Furuya M, Kanie Y, Imai H, Kita K, Uemura K, Okada S. A comparison between GPT-3.5, GPT-4, and GPT-4V: can the large language model (ChatGPT) pass the Japanese Board of Orthopaedic Surgery Examination? Cureus 2024;16:e56402. https://doi.org/10.7759/cureus.56402

41. Nicikowski J, Szczepanski M, Miedziaszczyk M, Kudlinski B. The potential of ChatGPT in medicine: an example analysis of nephrology specialty exams in Poland. Clin Kidney J 2024;17:sfae193. https://doi.org/10.1093/ckj/sfae193

42. Noda R, Izaki Y, Kitano F, Komatsu J, Ichikawa D, Shibagaki Y. Performance of ChatGPT and Bard in self-assessment questions for nephrology board renewal. Clin Exp Nephrol 2024;28:465-469. https://doi.org/10.1007/s10157-023-02451-w

43. Ozeri DJ, Cohen A, Bacharach N, Ukashi O, Oppenheim A. Performance of ChatGPT in Israeli Hebrew Internal Medicine National Residency Exam. Isr Med Assoc J 2024;26:86-88.

44. Panthier C, Gatinel D. Success of ChatGPT, an AI language model, in taking the French language version of the European Board of Ophthalmology examination: a novel approach to medical knowledge assessment. J Fr Ophtalmol 2023;46:706-711. https://doi.org/10.1016/j.jfo.2023.05.006

45. Patel JM, Hermann CE, Growdon WB, Aviki E, Stasenko M. ChatGPT accurately performs genetic counseling for gynecologic cancers. Gynecol Oncol 2024;183:115-119. https://doi.org/10.1016/j.ygyno.2024.04.006

46. Pham C, Govender R, Tehami S, Chavez S, Adepoju OE, Liaw W. ChatGPT’s performance in cardiac arrest and bradycardia simulations using the American Heart Association’s advanced cardiovascular life support guidelines: exploratory study. J Med Internet Res 2024;26:e55037. https://doi.org/10.2196/55037

47. Saad A, Iyengar KP, Kurisunkal V, Botchu R. Assessing ChatGPT’s ability to pass the FRCS orthopaedic part A exam: a critical analysis. Surgeon 2023;21:263-266. https://doi.org/10.1016/j.surge.2023.07.001

48. Sawamura S, Kohiyama K, Takenaka T, Sera T, Inoue T, Nagai T. Performance of ChatGPT 4.0 on Japan’s National Physical Therapist Examination: a comprehensive analysis of text and visual question handling. Cureus 2024;16:e67347. https://doi.org/10.7759/cureus.67347

49. Skalidis I, Cagnina A, Luangphiphat W, Mahendiran T, Muller O, Abbe E, Fournier S. ChatGPT takes on the European Exam in Core Cardiology: an artificial intelligence success story? Eur Heart J Digit Health 2023;4:279-281. https://doi.org/10.1093/ehjdh/ztad029

50. Smith J, Choi PM, Buntine P. Will code one day run a code?: performance of language models on ACEM primary examinations and implications. Emerg Med Australas 2023;35:876-878. https://doi.org/10.1111/1742-6723.14280

51. Song ES, Lee SP. Comparative analysis of the response accuracies of large language models in the Korean National Dental Hygienist Examination across Korean and English questions. Int J Dent Hyg 2025;23:267-276. https://doi.org/10.1111/idh.12848

52. Sood A, Mansoor N, Memmi C, Lynch M, Lynch J. Generative pretrained transformer-4, an artificial intelligence text predictive model, has a high capability for passing novel written radiology exam questions. Int J Comput Assist Radiol Surg 2024;19:645-653. https://doi.org/10.1007/s11548-024-03071-9

53. Stoehr F, Kampgen B, Muller L, Zufiria LO, Junquero V, Merino C, Mildenberger P, Kloeckner R. Natural language processing for automatic evaluation of free-text answers: a feasibility study based on the European Diploma in Radiology examination. Insights Imaging 2023;14:150. https://doi.org/10.1186/s13244-023-01507-5

54. Tarabanis C, Zahid S, Mamalis M, Zhang K, Kalampokis E, Jankelson L. Performance of publicly available large language models on Internal Medicine Board-style questions. PLOS Digit Health 2024;3:e0000604. https://doi.org/10.1371/journal.pdig.0000604

55. van Nuland M, Erdogan A, Aςar C, Contrucci R, Hilbrants S, Maanach L, Egberts T, van der Linden PD. Performance of ChatGPT on factual knowledge questions regarding clinical pharmacy. J Clin Pharmacol 2024;64:1095-1100. https://doi.org/10.1002/jcph.2443

56. Yang WH, Chan YH, Huang CP, Chen TJ. Comparative analysis of GPT-3.5 and GPT-4.0 in Taiwan’s medical technologist certification: a study in artificial intelligence advancements. J Chin Med Assoc 2024;87:525-530. https://doi.org/10.1097/JCMA.0000000000001092

57. Yoon SH, Oh SK, Lim BG, Lee HJ. Performance of ChatGPT in the In-Training Examination for anesthesiology and pain medicine residents in South Korea: observational study. JMIR Med Educ 2024;10:e56859. https://doi.org/10.2196/56859

**Not evaluated the performance of LLMs (13)**

1. Behrmann J, Hong EM, Meledathu S, Leiter A, Povelaitis M, Mitre M. Chat generative pre-trained transformer’s performance on dermatology-specific questions and its implications in medical education. J Med Artif Intell 2023;6:16. https://doi.org/10.21037/jmai-23-47

2. Lievin V, Hother CE, Motzfeldt AG, Winther O. Can large language models reason about medical questions? Patterns (N Y) 2024;5:100943. https://doi.org/10.1016/j.patter.2024.100943

3. Gilson A, Safranek CW, Huang T, Socrates V, Chi L, Taylor RA, Chartash D. How does ChatGPT perform on the United States Medical Licensing Examination (USMLE)?: the implications of large language models for medical education and knowledge assessment. JMIR Med Educ 2023;9:e45312. https://doi.org/10.2196/45312

4. Goenaga I, Atutxa A, Gojenola K, Oronoz M, Agerri R. Explanatory argument extraction of correct answers in resident medical exams. Artif Intell Med 2024;157:102985. https://doi.org/10.1016/j.artmed.2024.102985

5. Jaworski A, Jasinski D, Slawinska B, Blecha Z, Jaworski W, Kruplewicz M, Jasinska N, Syslo O, Latkowska A, Jung M. GPT-4o vs. human candidates: performance analysis in the Polish Final Dentistry Examination. Cureus 2024;16:e68813. https://doi.org/10.7759/cureus.68813

6. Mihalache A, Huang RS, Popovic MM, Muni RH. ChatGPT-4: an assessment of an upgraded artificial intelligence chatbot in the United States Medical Licensing Examination. Med Teach 2024;46:366-372. https://doi.org/10.1080/0142159X.2023.2249588

7. Sadeq MA, Ghorab RM, Ashry MH, Abozaid AM, Banihani HA, Salem M, Aisheh MT, Abuzahra S, Mourid MR, Assker MM, Ayyad M, Moawad MH. AI chatbots show promise but limitations on UK medical exam questions: a comparative performance study. Sci Rep 2024;14:18859. https://doi.org/10.1038/s41598-024-68996-2

8. Schmidgall S, Harris C, Essien I, Olshvang D, Rahman T, Kim JW, Ziaei R, Eshraghian J, Abadir P, Chellappa R. Evaluation and mitigation of cognitive biases in medical language models. NPJ Digit Med 2024;7:295. https://doi.org/10.1038/s41746-024-01283-6

9. Singhal K, Azizi S, Tu T, Mahdavi SS, Wei J, Chung HW, Scales N, Tanwani A, Cole-Lewis H, Pfohl S, Payne P, Seneviratne M, Gamble P, Kelly C, Babiker A, Scharli N, Chowdhery A, Mansfield P, Demner-Fushman D, Aguera Y Arcas B, Webster D, Corrado GS, Matias Y, Chou K, Gottweis J, Tomasev N, Liu Y, Rajkomar A, Barral J, Semturs C, Karthikesalingam A, Natarajan V. Large language models encode clinical knowledge. Nature 2023;620:172-180. https://doi.org/10.1038/s41586-023-06291-2

10. Stengel FC, Stienen MN, Ivanov M, Gandia-Gonzalez ML, Raffa G, Ganau M, Whitfield P, Motov S. Can AI pass the written European Board Examination in Neurological Surgery?: ethical and practical issues. Brain Spine 2024;4:102765. https://doi.org/10.1016/j.bas.2024.102765

11. Strong E, DiGiammarino A, Weng Y, Basaviah P, Hosamani P, Kumar A, Nevins A, Kugler J, Hom J, Chen JH. Performance of ChatGPT on free-response, clinical reasoning exams. MedRxiv [Preprint] 2023 Mar 29. https://doi.org/10.1101/2023.03.24.23287731

12. Valdez D, Bunnell A, Lim SY, Sadowski P, Shepherd JA. Performance of progressive generations of GPT on an exam designed for certifying physicians as certified clinical densitometrists. J Clin Densitom 2024;27:101480. https://doi.org/10.1016/j.jocd.2024.101480

**Insufficient results (13)**

1. Bicknell BT, Butler D, Whalen S, Ricks J, Dixon CJ, Clark AB, Spaedy O, Skelton A, Edupuganti N, Dzubinski L, Tate H, Dyess G, Lindeman B, Lehmann LS. ChatGPT-4 omni performance in USMLE disciplines and clinical skills: comparative analysis. JMIR Med Educ 2024;10:e63430. https://doi.org/10.2196/63430

2. Epstein RH, Dexter F. Variability in large language models’ responses to medical licensing and certification examinations: comment on “How does ChatGPT perform on the United States Medical Licensing Examination?: the implications of large language models for medical education and knowledge assessment”. JMIR Med Educ 2023;9:e48305. https://doi.org/10.2196/48305

3. Kao YS, Chuang WK, Yang J. Use of ChatGPT on Taiwan’s Examination for medical doctors. Ann Biomed Eng 2024;52:455-457. https://doi.org/10.1007/s10439-023-03308-9

4. Kelloniemi M, Koljonen V. AI did not pass Finnish plastic surgery written board examination. J Plast Reconstr Aesthet Surg 2023;87:172-179. https://doi.org/10.1016/j.bjps.2023.10.059

5. Kipp M. From GPT-3.5 to GPT-4. o: a leap in AI’s medical exam performance. Information 2024;15:543. https://doi.org/10.3390/info15090543

6. E K, S P, R G, R KL, A B, M G, T O, S R, V R, H M, G S. Advantages and pitfalls in utilizing artificial intelligence for crafting medical examinations: a medical education pilot study with GPT-4. BMC Med Educ 2023;23:772. https://doi.org/10.1186/s12909-023-04752-w

7. Knoedler L, Knoedler S, Hoch CC, Prantl L, Frank K, Soiderer L, Cotofana S, Dorafshar AH, Schenck T, Vollbach F, Sofo G, Alfertshofer M. In-depth analysis of ChatGPT’s performance based on specific signaling words and phrases in the question stem of 2377 USMLE step 1 style questions. Sci Rep 2024;14:13553. https://doi.org/10.1038/s41598-024-63997-7

8. Kung TH, Cheatham M, Medenilla A, Sillos C, De Leon L, Elepano C, Madriaga M, Aggabao R, Diaz-Candido G, Maningo J, Tseng V. Performance of ChatGPT on USMLE: potential for AI-assisted medical education using large language models. PLOS Digit Health 2023;2:e0000198. https://doi.org/10.1371/journal.pdig.0000198

9. Laupichler MC, Rother JF, Grunwald Kadow IC, Ahmadi S, Raupach T. Large language models in medical education: comparing ChatGPT- to human-generated exam questions. Acad Med 2024;99:508-512. https://doi.org/10.1097/ACM.0000000000005626

10. Rodrigues Alessi M, Gomes HA, Lopes de Castro M, Terumy Okamoto C. Performance of ChatGPT in solving questions from the Progress Test (Brazilian National Medical Exam): a potential artificial intelligence tool in medical practice. Cureus 2024;16:e64924. https://doi.org/10.7759/cureus.64924

11. Tsang R. Practical applications of ChatGPT in undergraduate medical education. J Med Educ Curric Dev 2023;10:23821205231178449. https://doi.org/10.1177/23821205231178449

12. Wang X, Gong Z, Wang G, Jia J, Xu Y, Zhao J, Fan Q, Wu S, Hu W, Li X. ChatGPT performs on the Chinese National Medical Licensing Examination. J Med Syst 2023;47:86. https://doi.org/10.1007/s10916-023-01961-0

13. Zare S, Vafaeian S, Amini M, Farhadi K, Vali M, Golestani A. Comparing the performance of ChatGPT-3.5-Turbo, ChatGPT-4, and Google Bard with Iranian students in pre-internship comprehensive exams. Sci Rep 2024;14:28456. https://doi.org/10.1038/s41598-024-79335-w

**Full-text unavailable (9)**

1. Arfaie S, Sadegh Mashayekhi M, Mofatteh M, Ma C, Ruan R, MacLean MA, Far R, Saini J, Harmsen IE, Duda T, Gomez A, Rebchuk AD, Pingbei Wang A, Rasiah N, Guo E, Fazlollahi AM, Rose Swan E, Amin P, Mohammed S, Atkinson JD, Del Maestro RF, Girgis F, Kumar A, Das S. ChatGPT and neurosurgical education: a crossroads of innovation and opportunity. J Clin Neurosci 2024;129:110815. https://doi.org/10.1016/j.jocn.2024.110815

2. Ghanem D, Nassar JE, El Bachour J, Hanna T. ChatGPT earns American Board Certification in hand surgery. Hand Surg Rehabil 2024;43:101688. https://doi.org/10.1016/j.hansur.2024.101688

3. Kawahara T, Sumi Y. GPT-4/4V’s performance on the Japanese National Medical Licensing Examination. Med Teach 2025;47:450-457. https://doi.org/10.1080/0142159X.2024.2342545

4. Shang L, Xue M, Hou Y, Tang B. Can ChatGPT pass China’s national medical licensing examination? Asian J Surg 2023;46:6112-6113. https://doi.org/10.1016/j.asjsur.2023.09.089

5. Tsoutsanis P, Tsoutsanis A. Evaluation of large language model performance on the Multi-Specialty Recruitment Assessment (MSRA) exam. Comput Biol Med 2024;168:107794. https://doi.org/10.1016/j.compbiomed.2023.107794

6. Wang H, Wu W, Dou Z, He L, Yang L. Performance and exploration of ChatGPT in medical examination, records and education in Chinese: pave the way for medical AI. Int J Med Inform 2023;177:105173. https://doi.org/10.1016/j.ijmedinf.2023.105173

7. Wang T, Mainous AG, Stelter K, O'Neill TR, Newton WP. Performance evaluation of the generative pre-trained transformer (GPT-4) on the family medicine in-training examination. J Am Board Fam Med 2024;37:528-582. https://doi.org/10.3122/jabfm.2023.230433R1

8. Wise J. Sixty seconds on . . . ChatGPT and medical exams. BMJ 2024;384:q675. https://doi.org/10.1136/bmj.q675

9. Yaneva V, Baldwin P, Jurich DP, Swygert K, Clauser BE. Examining ChatGPT performance on USMLE sample items and implications for assessment. Acad Med 2024;99:192-197. https://doi.org/10.1097/ACM.0000000000005549
