# Supplementary material for: Performance of large language models in medical licensing examinations: a systematic review and meta-analysis
Source: J Educ Eval Health Prof. 2025 Nov 18;22:36. doi: 10.3352/jeehp.2025.22.36 (PMC12976628; doi:10.3352/jeehp.2025.22.36)
Supplement: Supplementary file 5 — Supplement 4. Additional details of the included studies. [file jeehp-22-36-suppl4.docx]

**Supplement 4.** Additional details of the included studies

| Study (year) | Country | Language | Source of questions | Question type |
| --- | --- | --- | --- | --- |
| Rodrigues Alessi et al. [23] (2024) | Brazil | Portuguese | Brazilian National Medical Exam | MCQs |
| Alfertshofer et al. (2024) | Germany | Italian | Italian National Medical Examination | MCQs |
| Aljindan et al. [46] (2023) | Saudi Arabia | Arabic | SMLE | MCQs |
| Bicknell et al. [36] (2024) | USA | English | Internal Exam | MCQs |
| Ebrahimian et al. [53] (2023) | Iran | English | Internal Exam | MCQs |
| Fang et al. [40] (2023) | China | Chinese | CNMLE | MCQs |
| Flores-Cohaila et al. [44] (2023) | Peru | Spanish | Peruvian National Licensing Medical Examination | MCQs |
| Funk et al. [12] (2024) | Germany | English | AMBOSS | MCQs |
| Garabet et al. [37] (2024) | USA | English | AMBOSS | MCQs |
| Guillen-Grima et al. [13] (2023) | Spain | Spanish | MIR | MCQs |
| Haze et al. [28] (2023) | Japan | Japanese | JNME | MCQs-single choice |
| Huang et al. [48] (2024) | Taiwan | Chinese | SPTEMD | MCQs |
| Jaworski et al. [14] (2024) | Poland | Polish | Polish Final Medical Examination | MCQs |
| Kleinig et al. [15] (2023) | Australia | English | Australian Medical Council licensing examination | MCQs |
| Knoedler et al. [25] (2024) | Germany | English | AMBOSS | MCQs |
| Kufel et al. [32] (2024) | Poland | Polish | Polish Final Medical Examination | MCQs-single choice |
| Lai et al. [50] (2023) | UK | English | UKMLA | Single choice |
| Lin et al. [49] (2024) | Taiwan | Chinese | Taiwan medical licensing exams | Single choice |
| Liu et al. [9] (2024) | Japan | Japanese | JNME | MCQs |
| Mackey et al. [38] (2024) | USA | English | AMBOSS | MCQs |
| Meo et al. [47] (2023) | Saudi Arabia | English | Internal Exam | MCQs |
| Meyer et al. [16] (2024) | Germany | German | German medical licensing examination | MCQs |
| Ming et al. [17] (2024) | China | Chinese | CNMLE | MCQs-single choice |
| Morreel et al. [51] (2024) | Belgium | English | Internal Exam | MCQs |
| Nakao et al. [30] (2024) | Japan | Japanese | JNME | MCQs |
| Rojas et al. [52] (2024) | Chile | Spanish | EUNACOM | MCQs |
| Roos et al. [27] (2023) | Germany | German | German medical licensing examination | MCQs |
| Shieh et al. [39] (2024) | USA | English | USMLE | MCQs |
| Siebielec et al. [33] (2024) | Poland | Polish | Final Polish Medical Examination | MCQs |
| Suwała et al. [34] (2024) | Poland | Polish | Final Polish Medical Examination | MCQs |
| Tanaka et al. [18] (2024) | Japan | Japanese | JNME | MCQs |
| Tong et al. [41] (2023) | China | Chinese | CNMLE | Single choice |
| Torres-Zegarra et al. [45] (2023) | Peru | Spanish | Peruvian National Licensing Medical Examination | MCQs |
| Wojcik et al. [35] (2024) | Poland | Polish | Final Polish Medical Examination | MCQs |
| Yanagita et al. [31] (2023) | Japan | Japanese | JNME | MCQs |
| Zong et al. [43] (2024) | China | Chinese | CNMLE | MCQs |

MCQ, multiple choice question; SMLE, Saudi Medical Licensing Exam; CNMLE, Chinese National Medical Licensing Examination; MIR, Spanish Medical Residency Entrance Exam; JNME, Japanese National Medical Examination; SPTEMD, Taiwanese Staged Senior Professional and Technical Examinations for Medical Doctors; UKMLA, United Kingdom Medical Licensing Assessment.
