# Supplementary figures and images for: Performance of large language models in medical licensing examinations: a systematic review and meta-analysis
Source: J Educ Eval Health Prof. 2025 Nov 18;22:36. doi: 10.3352/jeehp.2025.22.36 (PMC12976628; doi:10.3352/jeehp.2025.22.36)

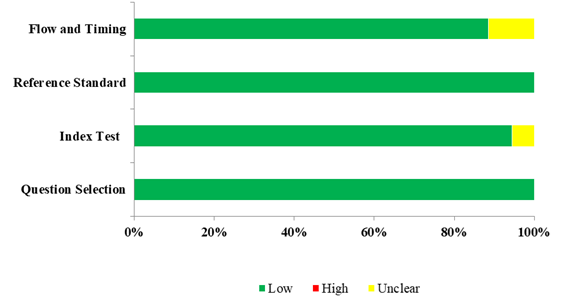


**Supplement 5.** Quality assessment of the included studies.

Supplement: Supplementary file 6 — Supplement 5. Quality assessment of the included studies. [file jeehp-22-36-suppl5.docx]

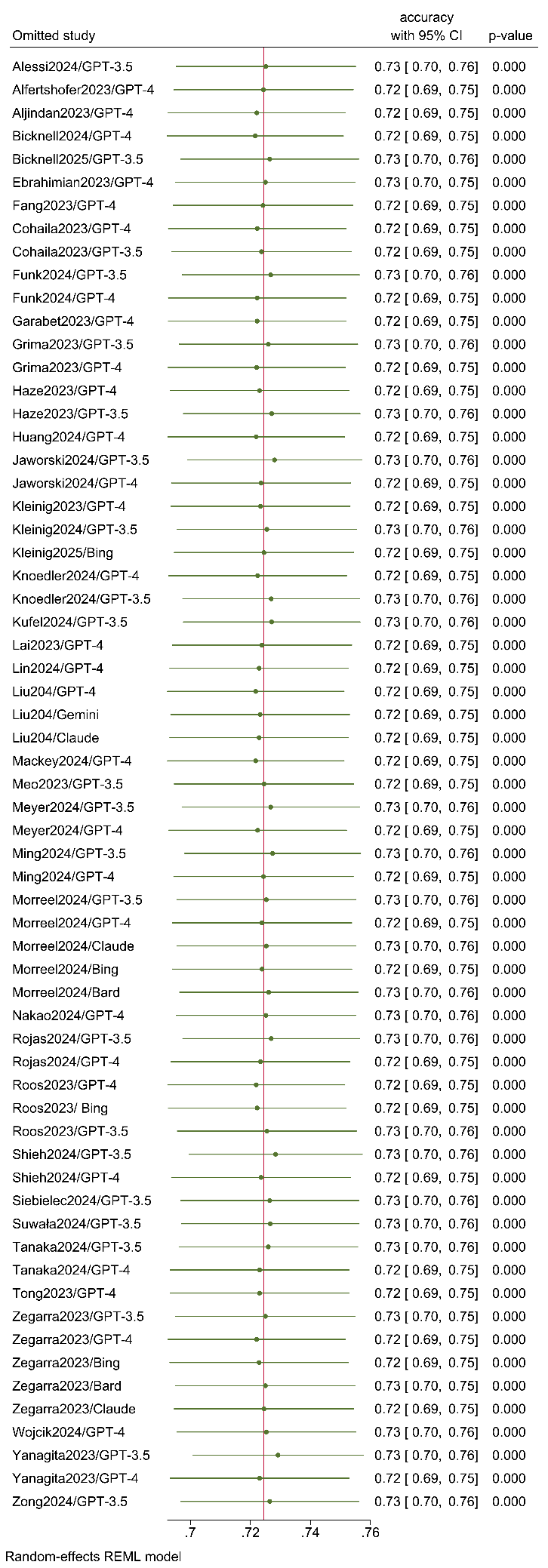


**Supplement 12.** leave-one-out meta-analysis. CI, confidence interval.

Supplement: Supplementary file 13 — Supplement 12. Leave-one-out meta-analysis. [file jeehp-22-36-suppl12.docx]

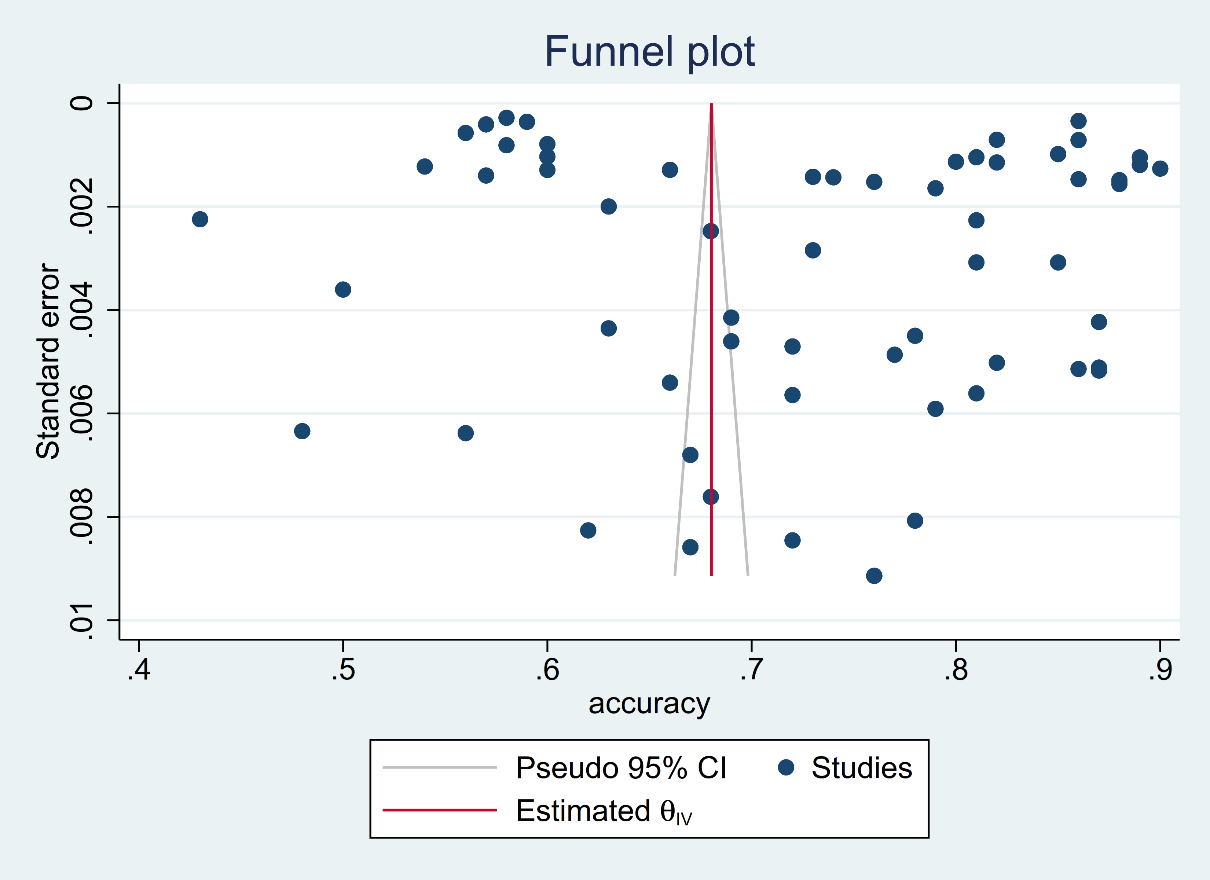


**Supplement 13.** Funnel plot for publication bias assessment. CI, confidence interval.

Supplement: Supplementary file 14 — Supplement 13. Funnel plot for publication bias assessment. [file jeehp-22-36-suppl13.docx]
