# Supplementary material for: Performance of large language models in medical licensing examinations: a systematic review and meta-analysis
Source: J Educ Eval Health Prof. 2025 Nov 18;22:36. doi: 10.3352/jeehp.2025.22.36 (PMC12976628; doi:10.3352/jeehp.2025.22.36)
Supplement: Supplementary file 7 — Supplement 6. The detailed information on the results of the quality assessment. [file jeehp-22-36-suppl6.docx]

**Supplement 6.** The detailed information on the results of the quality assessment

| Study (year) | Patient selection | Index test | Reference standard | Flow and timing |
| --- | --- | --- | --- | --- |
| Rodrigues Alessi et al. [23] (2024) | Low | Low | Low | Low |
| Alfertshofer et al. (2024) | Low | Low | Low | Low |
| Aljindan et al. [46] (2023) | Low | Low | Low | Low |
| Bicknell et al. [36] (2024) | Low | Low | Low | Unclear |
| Ebrahimian et al. [53] (2023) | Low | Low | Low | Low |
| Fang et al. [40] (2023) | Low | Low | Low | Unclear |
| Flores-Cohaila et al. [44] (2023) | Low | Low | Low | Low |
| Funk et al. [12] (2024) | Low | Low | Low | Low |
| Garabet et al. [37] (2024) | Low | Low | Low | Low |
| Guillen-Grima et al. [13] (2023) | Low | Unclear | Low | Low |
| Haze et al. [28] (2023) | Low | Low | Low | Low |
| Huang et al. [48] (2024) | Low | Low | Low | Low |
| Jaworski et al. [14] (2024) | Low | Low | Low | Low |
| Kleinig et al. [15] (2023) | Low | Low | Low | Low |
| Knoedler et al. [25] (2024) | Low | Low | Low | Low |
| Kufel et al. [32] (2024) | Low | Low | Low | Low |
| Lai et al. [50] (2023) | Low | Low | Low | Low |
| Lin et al. [49] (2024) | Low | Unclear | Low | Low |
| Liu et al. [9] (2024) | Low | Low | Low | Low |
| Mackey et al. [38] (2024) | Low | Low | Low | Low |
| Meo et al. [47] (2023) | Low | Low | Low | Unclear |
| Meyer et al. [16] (2024) | Low | Low | Low | Low |
| Ming et al. [17] (2024) | Low | Low | Low | Low |
| Morreel et al. [51] (2024) | Low | Low | Low | Unclear |
| Nakao et al. [30] (2024) | Low | Low | Low | Low |
| Rojas et al. [52] (2024) | Low | Low | Low | Low |
| Roos et al. [27] (2023) | Low | Low | Low | Low |
| Shieh et al. [39] (2024) | Low | Low | Low | Low |
| Siebielec et al. [33] (2024) | Low | Low | Low | Low |
| Suwała et al. [34] (2024) | Low | Low | Low | Low |
| Tanaka et al. [18] (2024) | Low | Low | Low | Low |
| Tong et al. [41] (2023) | Low | Low | Low | Low |
| Torres-Zegarra et al. [45] (2023) | Low | Low | Low | Low |
| Wojcik et al. [35] (2024) | Low | Low | Low | Low |
| Yanagita et al. [31] (2023) | Low | Low | Low | Low |
| Zong et al. [43] (2024) | Low | Low | Low | Low |
