# Supplementary material for: Performance of large language models in medical licensing examinations: a systematic review and meta-analysis
Source: J Educ Eval Health Prof. 2025 Nov 18;22:36. doi: 10.3352/jeehp.2025.22.36 (PMC12976628; doi:10.3352/jeehp.2025.22.36)
Supplement: Supplementary file 11 — Supplement 10. Subgroup analysis of LLM accuracy based on question format. [file jeehp-22-36-suppl10.docx]

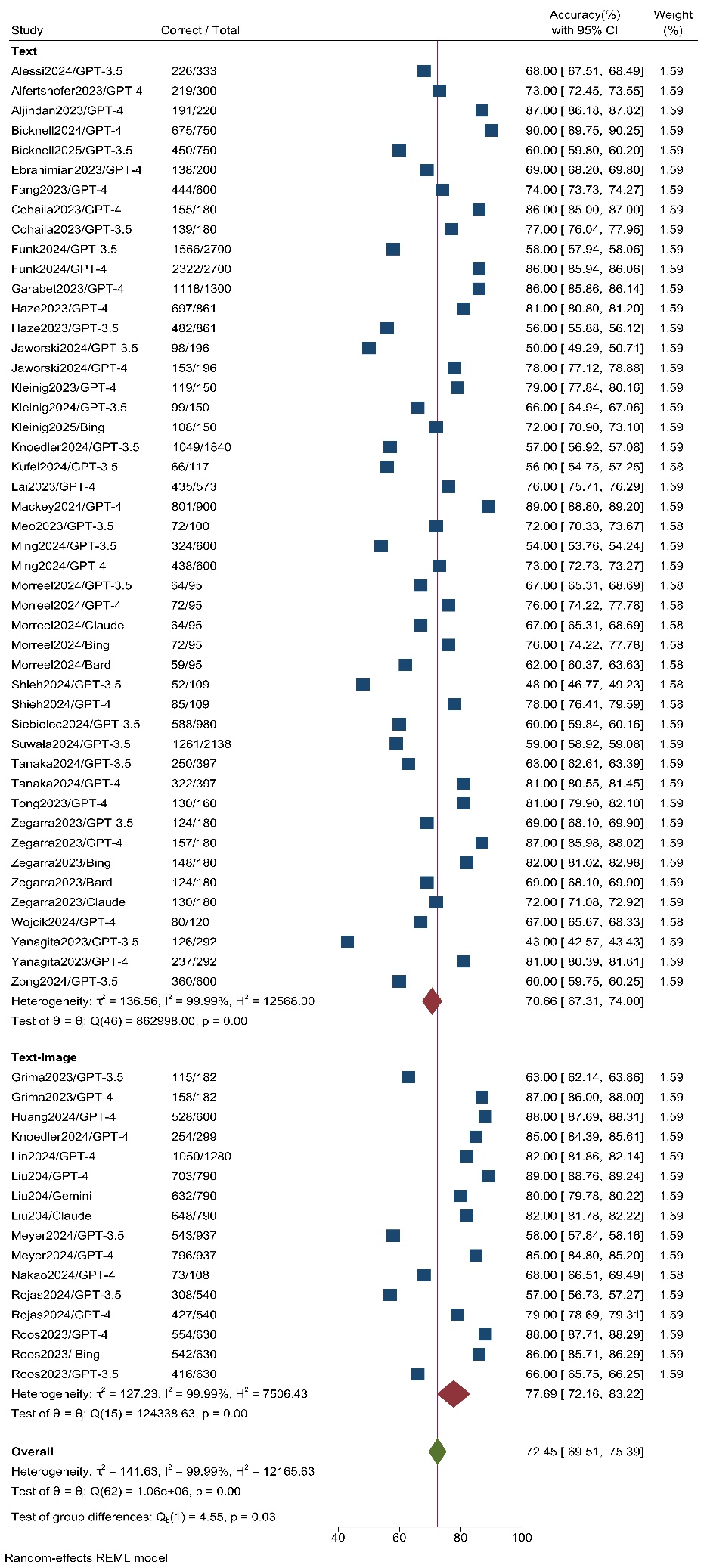


**Supplement 10.** Subgroup analysis of large language model (LLM) accuracy based on question format. CI, confidence interval.
